# Feasibility and reliability of an online version of the beat alignment test in neurotypical adults and people with stroke

**DOI:** 10.1186/s12938-025-01352-1

**Published:** 2025-02-24

**Authors:** Sarah Gregor, Avril Mansfield, George Mochizuki, Joyce Chen, Kara K. Patterson

**Affiliations:** 1 School of Rehabilitation Science, McMaster University, Hamilton, Canada; 2 Centre for Advancing Collaborative Healthcare & Education, University of Toronto, Toronto, Canada; 3 KITE-Toronto Rehabilitation Institute, University Health Network, Toronto, Canada; 4 Rehabilitation Sciences Institute, University of Toronto, Toronto, Canada; 5 Department of Physical Therapy, University of Toronto, 160-500 University Ave, Toronto, ON M5G1V7 Canada; 6 Evaluative Clinical Sciences, Hurvitz Brain Sciences Program, Sunnybrook Research Institute, Toronto, Canada; 7 School of Kinesiology and Health Science, York University, Toronto, Canada; 8 Faculty of Kinesiology and Physical Education, University of Toronto, Toronto, Canada

**Keywords:** Stroke, Music therapy, Musical rhythm, Perception, Test–retest reliability, Feasibility studies

## Abstract

**Background:**

Rhythm-based rehabilitation interventions are gaining attention and measuring their effects is critical. With more clinical care and research being conducted online, it is important to determine the feasibility of measuring rhythm abilities online. However, some tools used to measure rhythm abilities, in particular the beat alignment test (BAT), have not been validated for online delivery. This study aims to determine the feasibility, reliability, and learning effects for online delivery of the BAT in adults with and without stroke.

**Methods:**

Neurotypical adults and adults with chronic stroke completed the BAT online three times, with testing sessions separated by 2 to 4 days. The BAT includes a perception task (identifying whether tones overlayed on music matched the beat of the music) and a production task (tapping to the beat of music). Feasibility was evaluated with completion rates, technical challenges and resolutions, participant experience via exit questionnaire, and test duration. Reliability was measured using inter-class correlations and standard error of measurement, and learning effects were determined using a repeated-measures ANOVA.

**Results:**

Thirty-nine neurotypical adults and 23 adults with stroke participated in this study. More a priori feasibility criteria for the online BAT were met with neurotypical adults than people with stroke. Most components of the online BAT were considered reliable based on an ICC = 0.60 cut-off, except for perception in the neurotypical group, and production asynchrony in the stroke group. There was notable variability in performance, but no learning effects in either group.

**Conclusions:**

Online administration of the BAT is more feasible for neurotypical adults than people with stroke. Challenges with online administration for people with stroke may be partly related to the delivery platform. The BAT is a reliable tool with no learning effects and therefore is a promising way to assess for rhythm abilities online with careful consideration of user interface for people with stroke.

## Background

Stroke is a leading cause of adult disability in Canada and worldwide [1]. One promising approach to address this disability is using music as a tool to enhance neurological rehabilitation [2, 3]. For example, rhythmic auditory stimulation (RAS) for gait post-stroke involves walking to a rhythmic cue provided via music or a metronome [4]. Large effect sizes for speed, cadence, and stride length were confirmed by a meta-analysis of RAS interventions post-stroke [5]. RAS capitalizes on the extensive connectivity between the motor and auditory systems, which facilitates entrainment between the rhythmic auditory cues and motor responses while walking [6]. However, despite favorable changes in gait with RAS observed at the group level, there is variation in responsiveness to RAS at the individual level, and rhythm abilities may play a role [7, 8].

Rhythm is the pattern of silence, sound and emphasis found in music. The beat is the steady, regular pulse perceived in music [7]. When we feel the impulse to move to music, we usually tap to the beat rather than the rhythm [7]. This spontaneous synchronized movement is thought to arise from the processing of the musical beat in motor areas of the brain, specifically the supplementary motor area and basal ganglia (even when no movement is produced) [9]. An individual’s baseline rhythm abilities, and more specifically beat perception abilities mediate their response to rhythm-based rehabilitation interventions like RAS [8, 10]. For example, young neurotypical adults with weak beat perception walk with shorter steps and slower speed with RAS compared to those with strong beat perception [10]. This has implications for the use of RAS in people with stroke because their ability to perceive a beat is impaired compared to neurotypical older adults [7]. Furthermore, people with stroke who exhibit worse temporal gait asymmetry with RAS, had weaker beat perception compared to those who did not exhibit a worsening gait pattern with RAS [8]. Therefore, measuring beat processing abilities may help determine who is best suited for a particular music- or rhythm-based intervention. By identifying patients with weak beat perception and production abilities, a therapist can rule out RAS as an option and spend valuable [11] rehabilitation time on interventions that will be more effective for that patient.

The beat alignment test (BAT) is a test of musical beat perception and synchronization [12]. The BAT was designed to be a “naturalistic and simple, yet comprehensive” test of beat processing abilities in the general population [12]. Thus the BAT is easily understood by people with limited music background [13] and has been used with younger adults [14], older adults [7], and people with neurological diagnoses including Parkinson’s disease [11] and stroke [7]. Thus, the BAT holds promise as a targeted test of beat perception and synchronization to screen for the appropriateness of rhythm- and music-based rehabilitation interventions. There are two main components of the BAT: (1) a perception task, in which participants are asked to determine if tones superimposed on the music are congruent with the beat of the music; and (2) a production task, in which participants are asked to tap to the beat of the music [13]. Other tools that have been used to measure perceptual and sensorimotor timing and rhythm abilities include the Battery for the Assessment of Auditory Sensorimotor and Timing Abilities (BAASTA) [15], the Harvard Beat Assessment Test [16], and the computerized adaptive beat alignment test (CA-BAT) [17]. All these rhythm assessments include perception and production tasks similar to the BAT but, also include more complex tasks or a greater number of variations than the BAT. For example, compared to the single task of tapping to music stimuli in the BAT, the BAASTA has multiple tapping tasks including as tapping without a pacing stimulus, tapping to a metronome, tapping to music, synchronization continuation (start tapping to a pacing stimulus and continue tapping after the stimulus stops), and adaptive tapping (adapt tapping to the changing tempo in the pacing stimulus) [15]. Several factors limit the utility of these other assessments in the rehabilitation setting. For instance, the BAASTA can take 2.5–3 h to complete, the Harvard Beat Assessment requires specialized equipment, and the CA-BAT is adaptive, meaning that the difficulty of the tasks changes as the person progresses through the test making it harder to compare across participants. Therefore, it is likely that the BAT is the rhythm assessment most easily used for stroke rehabilitation research.

Any patient assessment, including tests of rhythm abilities, needs to adapt to a changing healthcare system. For example, there is a growing interest in the use of technology for the remote delivery of stroke rehabilitation (i.e., telerehabilitation) [18, 19]. Telerehabilitation is a feasible, effective, and acceptable alternative to in-person rehabilitation [20]. If rhythm-based interventions may be administered through telerehabilitation, and assessing rhythm abilities is important to understand who may respond best to such interventions, then it is also important to understand the feasibility of administering rhythm assessments online. The BAT is typically delivered in person with a researcher sitting beside the participant to answer any questions. Additionally, if the BAT is used as an outcome measure in research or clinical care, it is important to understand the psychometric properties and the potential practice effects when it is administered multiple times. Furthermore, components of validity and reliability have been assessed for the CA-BAT [17] and BAASTA [21], but not for the BAT.

The aim of this study is to (1) determine the feasibility of delivering the BAT online; (2) evaluate the reliability of an online version of the BAT in neurotypical adults and adults with stroke; and (3) evaluate practice effects of the online version of the BAT in neurotypical adults and adults with stroke.

## Results

### Participants

Demographic information for all participants, including musical background can be found in Table 1. Thirty-nine neurotypical adults and 23 adults with chronic stroke consented to participate in our study. Overall, a larger proportion of participants in the stroke group were considered older adults (≥ 60 years old): neurotypical 13% (5/39), stroke 40% (9/22). Both groups had primarily women with 56% (22/39) and 83% (19/23) women in the neurotypical and stroke group, respectively. More participants in the neurotypical group reported formal music training (19/39, 49% neurotypical, and 10/23, 44% stroke), and informal music training (16/39, 41% neurotypical, and 6/23, 26% stroke). Informal music training was mostly described as participants teaching themselves a musical instrument. One person with stroke did not report their music experience.

**Table 1:** Online BAT participant demographics

	Neurotypical (*n =* 35)^a^	Chronic stroke (*n =* 23)
Age (*n*, %)		
18–29 years	16 (46)	1 (4)
30–39 years	7 (20)	1 (4)
40–49 years	3 (9)	4 (17)
50–59 years	4 (11)	7 (30)
60–69 years	3 (9)	7 (30)
≥ 70 years	2 (6)	2 (9)
NR	0 (0)	1 (4)
Gender (*n*, %)		
Man	13 (37)	4 (17)
Woman	22 (63)	19 (83)
Stroke characteristics		
Time post-stroke, (months, SD)	N/A	110(135)
Side of paresis (*n*, %)		
Left		11 (48)
Right		6 (26)
Both		2 (9)
Unknown		2 (9)
NR		2 (9)
Type of stroke (*n*, %)		
Ischemic		13 (57)
Hemorrhagic		5 (22)
Unknown		4 (17)
NR		1 (4)
Reported music experience^a^		
Formal in school (*n*, %) Duration of experience (years, SD)	13 (37.1)	10 (45.5)
	1.3 (2.2)	1.9 (3.2)
Formal outside of school (n, %) Duration of experience (years, SD)	15 (42.9)	3 (13.6)
	2.8 (4.0)	1.3 (3.4)
Informal music experience (n, %)	16 (45.7)	6 (27.3)
GSI (score/7, SD)^a^		
Active engagement	3.7 (1.3)	3.3 (1.2)
Perceptual abilities	5.0 (0.8)	4.8 (0.9)
Musical training	3.4 (1.7)	2.2 (1.2)
Singing abilities	3.7 (1.0)	3.2 (1.1)
Emotions	4.9 (1.3)	4.8 (1.2)
General sophistication	3.8 (1.2)	3.2 (0.9)

Values presented are means with standard deviations in parentheses (continuous variables) or counts with percentages in parentheses (categorical variables)

GSI, Goldsmith sophistication index; NR, not reported; N/A, not applicable; SD, standard deviation

^a^35 neurotypical participants completed the demographics and music background, 1 participant with stroke did not answer questions related to their music background

### Feasibility

#### Completion rates

**Fig. 1:**
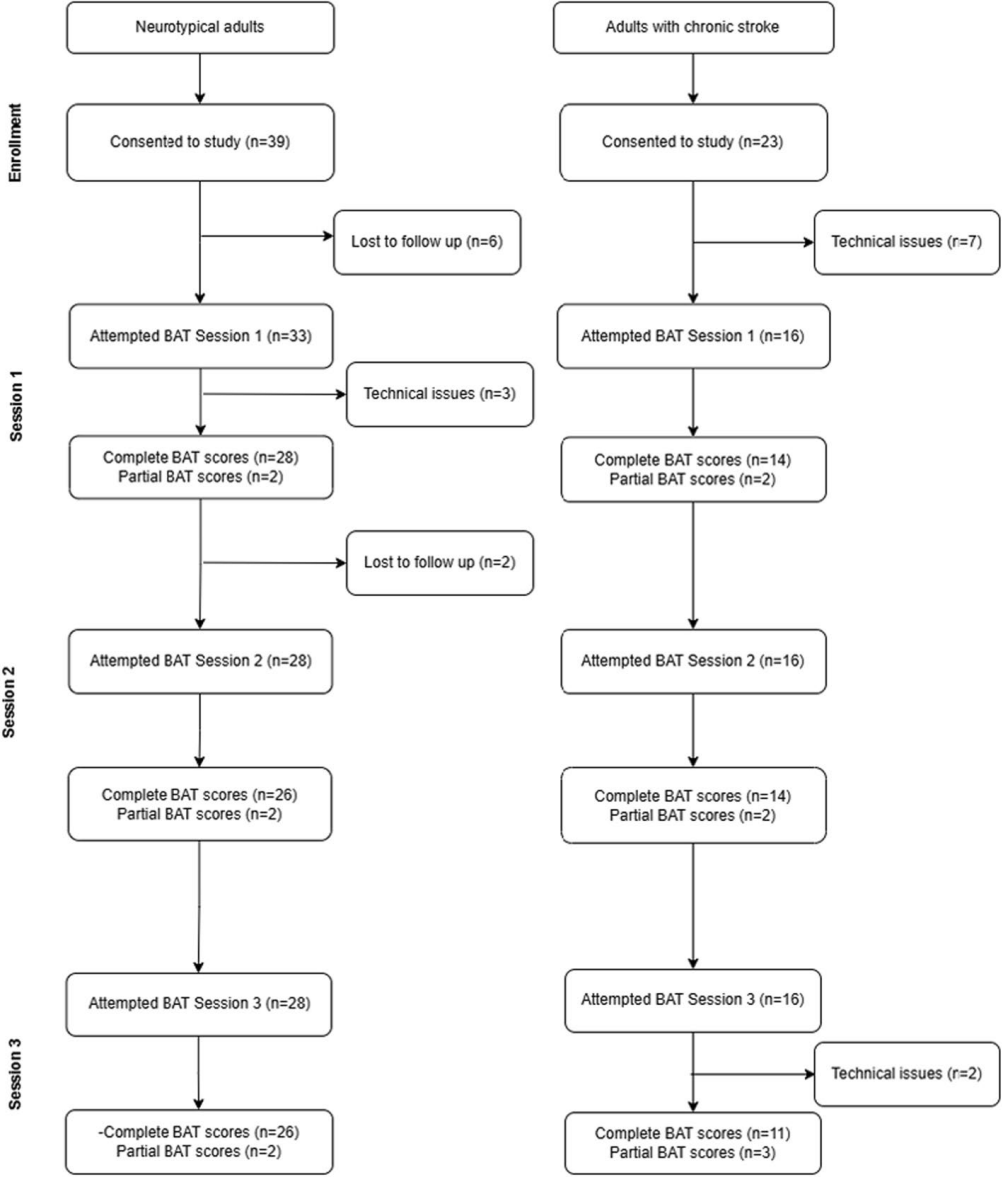
Flowchart of completion of BAT by participants at each session. Participants lost to follow-up had no recorded attempt of the BAT and did not respond to the investigator’s communication attempts. “Technical issues” indicates participants that attempted the BAT but no BAT data was obtained. Complete BAT scores indicates participants for whom all 3 scores were obtained, and partial BAT scores indicates participants for whom at least 1 score was obtained for that session

Figure 1 illustrates the completion of the BAT test at each session for neurotypical adults and adults with chronic stroke. Twenty-eight neurotypical adults (28/39, 72%) and 14 (14/23, 61%) people with stroke attempted the BAT with some scores collected at all three sessions.

#### Technical issues and resolutions

Of the participants who consented (*n =* 39 neurotypical, *n =* 23 stroke), 14/39 (39%) neurotypical adults and 21/23 (91%) adults with stroke experienced technical issues and/or required assistance to complete the BAT. This rate of technical issues did not meet the a priori feasibility criterion of < 25% of participants. Four (80%) of 5 the neurotypical adults > 60 years old and 9 (100%) of the 9 adults with stroke > 60 years old required assistance. Of the participants who required assistance at any point during the study (*n =* 14 neurotypical and *n =* 21 stroke), the issues were either fully resolved so that all 3 BAT scores were recorded or partially resolved so that at least one of the 3 BAT scores were recorded for 5/14 (36%) of the neurotypical adults and 12/21 (57%) of the adults with stroke. This did not meet the a priori feasibility criterion of 75% resolution rate for technical difficulties. Technical challenges often resulted in a longer interval between BAT sessions (range 1–6 days); however, these data were included in the main analysis as a prolonged interval does not influence test–retest reliability [22].

Common technical difficulties reported were challenges downloading the BAT experiment, or difficulties returning to the JotForm page to complete the study after finishing the BAT. Common feedback reported about the BAT in the open-ended responses included issues with the volume, as one neurotypical participant reported *“I found the beats in the [perception] test so loud that I could not hear the music clearly”.* Issues with volume were reported by more people in the stroke group (*n =* 3) than in the neurotypical group (*n =* 1). One recommendation for the production component of the BAT was to improve the clarity about how they should tap to the beat “*I just realized with this second session of the production exercise that my tapping varies. Sometimes, I am tapping 1–2-3–4-1–2-3–4; sometimes, I am tapping 1-…-3-…-1-…-3-.. […] I assume that you are taking this into account”.* Other suggestions for the BAT were related to using different music clips for the practice trials than the experiment and having the option to re-do a trial (for example if the participant was distracted). Five participants in the neurotypical group and 6 participants in the stroke group reported not being confident that they did the experiment correctly.

Technical issues that were not resolved and the number of participants who experienced them included: (1) issues with the graphics display of the BAT on the computer screen (likely due to an older incompatible version of Windows software)—2 neurotypical adults, 5 people with stroke; (2) attempting the study on an incompatible computer (i.e., Apple)—1 neurotypical adult, 1 person with stroke; and (3) technical issue not reported—1 person with stroke.

One final issue was completing the same component of the BAT multiple times in a single study session (*n =* 2). One neurotypical participant completed the test twice at time point 1 and three times at time point 3. A second neurotypical participant completed the BAT twice at time point 2. This may be attributed to participants being unaware they repeatedly clicked the same link. In these cases, the scores from the first attempt were used in the analysis.

#### User experience and BAT duration

Table 2 summarizes the ratings for the 7 items on the exit questionnaire for each group. Ratings for all items met the a priori feasibility criterion (median rating = 4/5) except for Item 1 (“I found the rhythm perception test easy to do online) in the stroke group. The rating of BAT duration as “just right” also met the feasibility criterion in both groups.

**Table 2:** Responses to exit questionnaire

Exit questionnaire item	Feasibility threshold criterion	Neurotypical	Chronic stroke
1. I found the rhythm perception test easy to do online	Median rating ≥ 4/5	4 (1–5)	3 (1–5)
2. I found the rhythm production test easy to do online		4 (2–5)	4 (2–5)
3. I found the rhythm perception test instructions clear		5 (2–5)	4 (2–5)
4. I found the rhythm production test instructions clear		5 (2–5)	5 (4–5)
5. I found this test fun to do		5 (1–5)	4 (1–5)
6. I think this test accurately measured my rhythm perception abilities		4 (1–5)	4 (2–5)
7. I think this test accurately measured my rhythm production abilities		4 (1–5)	4 (2–5)
Rating of BAT duration (*n*, %)^a^	≥ 75% rated the BAT to be the right length		
Too short		2/32 (6%)	2/17 (12%)
Too long		3/32 (9%)	1/17 (6%)
Right length		27/32 (84%)	14/17 (82%)

Feasibility of the online BAT. Variables are presented as medians with ranges in parentheses or as frequency counts with percentages in parentheses

^a^Neurotypical *n =* 32, stroke *n =* 17 completed the exit questionnaire

### Reliability

Average BAT scores over time as well as the ICC, SEM, and MDC for each measure of the BAT in both groups are presented in Table 3. In the neurotypical group, the ICC for production component of the BAT (both the asynchrony and variability scores) exceeded the 0.60 minimum threshold for clinical tests. In the stroke group, the ICC for the perception component and the variability score for the production component exceeded the minimum threshold for clinical tests.

**Table 3:** Reliability of the BAT

Group	Time 1 score	Time 2 score	Time 3 score	ICC (3,1)^b^	SEM^a^	MDC
Perception accuracy (% correct)						
NT	64.27 (35.29–88.23)	63.18 (23.43–94.12)	66.74 (29.41–94.12)	0.50	6.42	17.79
Stroke	57.56 (35.29–82.35)	55.88 (23.53–94.12)	53.92 (29.41–88.24)	0.80	3.56	9.86
Production asynchrony (msec)						
NT	100.02 (82.07–137.01)	96.51 (71.31–132.01)	99.11 (78.41–133.76)	0.73	3.12	8.64
Stroke	99.13 (76.77–121.47)	99.48 (78.69–118.97)	102.50 (81.03–126.46)	0.47	5.16	14.30
Production variability (coefficient of variation, %)						
NT	10.77 (4.16–41.58)	11.49 (3.95–48.73)	9.47 (3.63–31.11)	0.89	0.78	2.16
Stroke	12.46 (4.89–21.35)	12.58 (4.37–23.76)	14.94 (5.83–46.97)	0.80	1.68	4.64

Values for rhythm perception and production scores for each group at each time point are presented as means with ranges in parentheses, with ICC, SEM, and MDC values also provided

NT, neurotypical; ICC, intra-class correlation; SEM, standard error of measurement; MDC, minimal detectable change; %, percent; ms, millisecond

^a^Average of 3 time points

^b^ICC calculated based on time points 1 and 2

### Learning effects

For the BAT perception task score (perception accuracy) there was no significant within-subject effect for time (F(2,70) = 0.73, *p =* 0.49, η_G_^2^ = 0.0053). There was a significant main effect for group (F(1,35) = 10.02, *p =* < 0.01, η_G_^2^ = 0.1650) but no significant group*time interaction (F(2,70) = 0.73, *p =* 0.49,η_G_^2^ = 0.0053). For the BAT production task asynchrony score there was no significant within-subject effect for time (F(2,78) = 0.65, *p =* 0.53, η_G_^2^ = 0.0035). There was also no significant main effect of group (F(1,39) = 0.36, *p =* 0.55, η_G_^2^ = 0.0184) nor was there a significant group*time interaction (F(2,78) = 1.26, *p =* 0.38, η_G_^2^ = 0.007). For the BAT production task variability score there was no significant within-subject effect of time (F(2,78) = 0.10, *p =* 0.90, η_G_^2^ = 0.0008), no significant effect of group (F(1,39) = 1.97, *p =* 0.17, η_G_^2^ = 0.05)and no significant group*time interaction (F(2,78) = 1.45, *p =* 0.24, η_G_^2^ = 0.0078).

## Discussion

This study assessed the feasibility, test–retest reliability, and learning effects of an online version of a commonly used test of rhythm abilities: the beat alignment test (BAT). Our study produced several interesting findings. First, online remote administration of the BAT did not meet all the feasibility criteria for neurotypical adults and was even less feasible for people with chronic stroke. This was mostly attributable to technical issues associated with the specific platform used to deliver the test. Second, there were no learning effects for the BAT in either neurotypical adults or adults with stroke, which facilitates its use as a repeated outcome measure. Third, as a clinical test, only one of the three BAT components, the variability score for the BAT production task, met the minimum ICC criterion (i.e., 0.60) in both neurotypical adults and adults with stroke. Other components of the BAT met the clinical criterion for only one of the two groups: the perception task for the stroke group and the production asynchrony score for the neurotypical group).

There are a few factors that may have contributed to the observed lower feasibility in the stroke group. First, based on proportions of participants in each age group, the stroke group was older. Previous work has noted that older adults (> 54 years old) have more challenges navigating telehealth websites than younger adults [23], even though older adults show similar interest and satisfaction in using technology in healthcare settings [24]. The BAT protocol in the present study required participants click on/close multiple windows, which may have caused difficulties for older participants in the stroke group. Moreover, all but one participant over the age of 60 in either group required help from the researchers to complete this study. Therefore, the present results suggest that age contributes to feasibility of testing online.

A second factor that could have led to lower feasibility is stroke-related cognitive impairment. Telemedicine is more successful with people with stroke when it is easier to use and matches an individual’s functional abilities (i.e., motor function and cognition) [25]. Cognition was not assessed in our study, as previous work found no relationship between BAT scores and cognition after stroke [7]. However, 40–79% of people with stroke have mild cognitive impairment [26, 27]. Therefore, it is possible that some participants with stroke in the present study had mild cognitive impairment. These individuals may have experienced difficulty navigating through the BAT online, even though cognition likely had limited direct impact on BAT scores.

A third factor that may have contributed to lower feasibility in the stroke group is “hidden or invisible” disabilities due to impaired visual and/or auditory perception. Vision impairments affect up to 60% of people with stroke [28], which could have impacted the ability of participants in the present study to complete the BAT. For example, visual field loss or visual inattention could have prevented them from properly scanning the computer screen and locating the correct window to click, resulting in incomplete BAT sessions. Furthermore, central auditory processing disorder is the most common type of hearing impairment in people with stroke (40–55%) [29]. Participant comments in the present study related to volume of the stimuli and difficulty distinguishing the tones from the music clip during the perception task may be partly attributed to this invisible disability. Future work should investigate how cognitive function and invisible visual and auditory disabilities contribute to the success of online testing of rhythm abilities after stroke. In addition, applying signal detection theory to the BAT perception score may provide additional insight into beat processing in people with stroke by separating the behavior into sensitivity and bias [30].

Overall, our ICC analysis showed that the reliability of the various scores generated by the BAT ranged from poor to good test–retest reliability in both groups, with some scores meeting the threshold for use as a clinical test. There was notable variability for each measure within both groups (based on the SEM analysis), especially rhythm perception and production asynchrony. The perception task specifically required the participant to pay attention to the auditory stimulus and only respond at the end of each trial. Research shows that younger participants commonly multi-task with online study surveys [31], and therefore may have been multitasking and distracted during our study. This may explain the high variability observed in the rhythm perception task, especially in the neurotypical group, which included younger participants.

Test–retest reliability in the present study was lower than other measures of rhythm abilities assessed in neurotypical populations. For example, a previous test–retest reliability study on the BAASTA (which used different musical stimuli than the BAT), reported perception score ICC = 0.94, rhythm production accuracy ICC = 0.77, and consistency ICC = 0.97 in 20 neurotypical older adults [21]. It is important to note that the perception score was calculated differently from the present study (number of hits (correct identification of tone off beat) / number of misses (inaccurate identification of a tone off beat)) [21]. This difference in test–retest reliability in neurotypical adults between the BAASTA and the BAT in the present study may be attributed to the BAASTA being a much longer test overall. A series of validation and test–retest studies on the CA-BAT found that increasing the length of the rhythm assessment improves test–retest reliability in young neurotypical adults (sample size ranged from *n =* 71 to = 223) [17]. Furthermore, the BAASTA was delivered in person. Rhythm testing in a laboratory setting produces better test–retest reliability because listening conditions are consistent between testing sessions [17]. In the present study, the online delivery of the BAT meant that beyond giving participants instructions, we had little control over the listening conditions. This also may have attributed to the lower reliability compared to the BAASTA.

Poorer reliability with online testing in the present study may be a result of participants not being able to ask clarifying questions in real-time. For example, participants reported not knowing if they should “tap to the whole note, half note, or quarter notes”, with some participants reporting switching their approach throughout the testing. While our calculation of production asynchrony and variability takes these different approaches into account, the uncertainty in instructions may have influenced participants’ confidence and therefore behaviors during the production task. Future work with online administration of the BAT should consider having research team members call participants while they complete the online test to help clarify instructions.

Recommendations by the Stroke Recovery and Rehabilitation Roundtable state that researchers developing and testing novel interventions should identify characteristics of individuals who respond best to ensure interventions are efficient and effective [32]. With rising interest in music- and rhythm-based interventions, and evidence that rhythm abilities may mediate who responds best to these interventions it is important to be able to accurately measure rhythm abilities. To our knowledge, this is the first study to evaluate the feasibility and test–retest reliability of a rhythm assessment for people with stroke in an online context. The online version of the BAT holds promise as a reliable test to measure rhythm abilities, however, work is needed to make the test more feasible for people with stroke. Future work should consider if completing the test online simultaneously with a live call with a researcher to provide step-by-step instructions and answer questions improves feasibility and increases test–retest reliability.

### Limitations

As participants completed this study in their homes, we were unable to control the environment (e.g., distractions, background noise) and technology (e.g., types of headphones) used during the study. For example, while we screened for hearing abilities as part of our eligibility criteria, we were unable to do a headphone test as recommended by Wood and colleagues for web-based auditory experiments [33]. Furthermore, we were unable to evaluate how much individuals paid attention to the task or whether there were any issues with sound quality that could have influenced BAT scores. Finally, demographic data were collected by self-report, and we were unable to confirm the diagnosis, type of stroke or lesion characteristics with medical records or imaging which are characteristics that may have influenced BAT performance.

## Conclusions

Repeated online administration of the BAT exhibited no learning effects and test–retest reliability ranged from poor to good for neurotypical adults and adult with stroke.

Previous in-person administration of the BAT with people with stroke has been successful [34]. However, future work is needed to improve online administration of the BAT. The influence of cognitive and perceptual deficits on online BAT performance should be examined, possibly by including an in-person visit in the protocol to collect demographics and assess impairments. Modifications to online delivery (e.g., administered in real-time with a researcher via web conferencing) to improve feasibility and test–retest reliability and assessment of these modifications with a validated usability measure (e.g., System Usability Scale [35]) are also required before clinical implementation is possible.

## Methods and materials

### Participants

This study included two groups of adult participants (over 18 years old): neurotypical adults and people with chronic (> 6 months) stroke). Inclusion criteria were self-reported (1) access to a computer with Windows software; (2) access to speakers (including those built into the computers); and (3) ability to understand written English. Participants were excluded if they had: (1) more than mild hearing loss (based on self-report); (2) diagnoses of neurological disorders (other than stroke); and/or (3) completed the BAT or another rhythm assessment within the last year. Participants were recruited using online advertising through physiotherapy clinics specializing in neurorehabilitation, multiple social media outlets (i.e., Twitter, Facebook), and through Heart and Stroke Foundation advertisements. All participants provided informed consent prior to participation in this study. This study was approved by the University of Toronto Research Ethics Board.

### Sample size calculation

Sample size calculation was derived from the formula of intra-class correlation coefficient (ICC) test as outlined by Bujang and coauthors [36]. When alpha and power are 0.05 and 80%, respectively, a minimum sample size of 15 is sufficient to detect an ICC of 0.60 when there are 2 observations [36]. We selected an ICC value of 0.60 because this has been described as the minimal acceptable ICC value for clinical evaluations [37]. Therefore, the minimal sample size for recruitment to power our ICC calculation is 15 people per group. We increased our recruitment goal by 50% to 23 per group to account for potential technical issues, missing data and participants withdrawal.

### Study protocol

All components of the study, including consent, eligibility assessment, and questionnaires were completed using the online platform JotForm (2021 JotForm Inc., San Francisco, USA). Links to the BAT experiments, which were completed using E-prime Go software (Psychology Software Tools, Pittsburgh, USA), were also provided on the JotForm webpage.

Study participants completed the BAT three times, each separated by 2–4 days, to give flexibility for participants who completed the study in their homes. The 2–4 day timeline was chosen as research suggests that when a population is clinically stable, an interval between tests longer than 2 days does not significantly change test–retest reliability [22].

#### Demographics

Prior to completing the first session of the BAT, participants completed a demographic questionnaire to collect age, gender identity, and previous experience completing the BAT. If applicable, participants also answered close-ended questions about their stroke including type (i.e., what type of stroke did you have? Ischemic, hemorrhagic, unknown) and hemiparesis (i.e., what side of your body was affected? Right, left, both, unknown). Participants also completed a questionnaire about formal and informal music training and completed the Goldsmith musical sophistication index (GSI) [38, 39]. Musical sophistication is defined as music behaviors, skills, experiences, and achievements of an individual [38]. The GSI includes questions that span 6 sub-categories (active engagement, perceptional abilities, musical training, singing abilities, emotions, and general sophistication), which explore how people engage with music in Western society [38].

#### BAT perception and production tasks

The BAT has 2 components: the perception task and the production task. Participants watched an instructional video that replaced the verbal instructions usually provided with in-person administration of the BAT. The instruction video was embedded into the JotForm page and included information regarding how to download the experiment from E-Prime Go, run the experiment, and get back to the JotForm page to continue with the study. These instructions were also provided in written text followed by the link to the E-Prime Go download page. Participants were instructed to complete the BAT in a quiet space using headphones.

The online version of the BAT included the same instructions and stimuli typically used with in-person administration. To adapt for delivery online, we provided participants the opportunity to repeat practice trials if they felt they did not understand the experiment. The perception task of the BAT required participants to determine if tones overlaid on music were on or off the beat of the song [13]. For the off-beat trials, the tones were either (a) too fast/slow in tempo relative to the music (tempo error); or (b) out of phase with the actual beat of the music (phase error) [13]. The response options to the prompt “are the tones on the beat of the music?” were yes or no. There were 17 musical excerpts with an average duration of 16 s [13]. Perception was evaluated as accuracy of responses; the percentage of trials that participants accurately determined if the tone was on or off the beat of the music (perception accuracy (%) = number of trials correct/17 *100).

The BAT production task required participants to tap to the beat of a song with the keyboard space bar (stroke participants used their unaffected hand). The same musical excerpts were used for both perception and production tasks [13]. Rhythm production ability was quantified in two ways: (1) asynchrony—the difference in time between stimulus onset and the participant’s taps in milliseconds, averaged across all trials; and (2) variability—the variability in inter-tap intervals (ITI) that is the timing between participant taps (coefficient of variation (%) = standard deviation ITI/mean ITI * 100).

#### Feasibility

The present study was guided by work on feasibility studies by Bowen and colleagues [40] and previous work that evaluated online arts-based therapies in people with stroke [41]. Bowen colleagues outlined the purpose of feasibility studies as: “to identify not only what—if anything—in the research methods or protocols needs modification but also how changes might occur” [40]. Of the 8 areas of focus addressed by feasibility studies, two applied to the current work: implementation and acceptability [40]. Domains within these two areas of focus that were identified as important for administering the BAT remotely were as follows:

*Implementation* (to what extent can a measure be successfully delivered to intended participants? [40]): BAT completion rate (percentage of consenting participants who completed all 3 test sessions), technical issues/requests for assistance (number and type), and resolution of technical issues (description of solution and resolve rate measured as number of issues/requests resolved/total number issues/requests). A participant was determined to have complete data at a session if all BAT data (i.e., 3 scores) were recorded and partial data if at least 1/3 BAT scores were recorded. These parameters were tracked throughout the study.

*Acceptability* (to what extent is a measure judged as suitable, satisfying or attractive to recipients? [40]): suitability and satisfaction were measured with an exit questionnaire administered after participants completed the BAT for the first time. The exit questionnaire consisted of 7 items related to suitability and satisfaction with the online BAT (e.g., clarity of instructions, ease of use, enjoyment, etc.). Participants rated their agreement with the 7 items using a 5-point Likert scale (1 = strongly disagree to 5 = strongly agree). Participants also rated the length of the BAT as too short, the right length, or too long. Finally, participants were requested to provide any other comments about technical difficulties or recommendations to improve this test online.

### Statistical analysis

#### Demographics

Demographic data were presented with descriptive statistics, including mean and standard deviation for continuous data (i.e., time post-stroke, rhythm abilities) and frequency counts and percentages for categorical and ordinal data (i.e., age, group, and gender).

#### Objective 1: feasibility

Parameters for each of the feasibility domains were analyzed with descriptive statistics for both study groups. The threshold for feasibility was determined for each domain based on what we estimated to be clinically important: (a) completion rate: ≥ 75% of participants complete all 3 sessions; (b) technical issues: ≤ 25% of participants report technical issues; (c) technical issue resolution: ≥ 75% of all issues are resolved; (d) user experience: all items on the exit questionnaire have a median rating of ≥ 4/5; and (e) BAT length: ≥ 75% of participants rate the BAT as the ‘right length’. The proportion of older adults (i.e., ≥ 60 years old, as defined by the United Nations [42]) that reported technical challenges during this study was also calculated. A qualitative descriptive analysis was used for any open-ended responses.

#### Objective 2, 3: reliability and learning effects

Data for neurotypical adults and people with stroke were analyzed separately. We followed the guidelines for the selection and reporting of intra-class correlation coefficients (ICC) as outlined by Koo and Li [43]. To determine the test–retest variability of sessions 1 and 2, a two-way mixed effects ICC with a single rater/measurement (ICC 3,1) analysis was computed with the BAT perception, BAT production asynchrony, and BAT production variability scores as the dependent variable [43]. Therefore 3 ICCs were computed each for the stroke and neurotypical groups. The ICC value was interpreted as poor (< 0.50), moderate (0.5–0.75), good (0.75–0.9), and excellent (> 0.90) based on the recommendations of Koo and Li (2016) [43]. An ICC of 0.60 is the minimum required for clinical tests [37], and therefore was set a priori as the threshold for reliability of the online BAT.

To determine the precision and expected variability of each BAT score, the standard error of measurement (SEM) was calculated (SEM = SD*√(1-ICC)) [44, 45]. The SEM can be thought of as a measure of absolute reliability (i.e., consistency of the scores of individuals) [46], and was used to determine the minimal detectable change (MDC = SEM*1.96* √2) [46] each group for each measure of the BAT.

To determine learning effects when doing the online BAT, a 2 × 3 (group x time) repeated-measures analysis of variance (ANOVA), was used to compare all time points of BAT scores within both groups. The independent variable was group (i.e., stroke or neurotypical) and the dependent variable was BAT score (i.e., perception accuracy, production asynchrony, production variability). Significant within-subject main effects of time (p < 0.05) were interpreted as evidence of learning. The generalized eta squared (η_G_^2^) was calculated for between and within-subject effects as a measure of effect size that is comparable across different research designs [47, 48].

## Data Availability

No datasets were generated or analyzed during the current study.

## Abbreviations

ANOVA: Analysis of variance
BAASTA: Battery for the Assessment of Auditory Sensorimotor and Timing Abilities
BAT: Beat alignment test
CA-BAT: Computerized adaptive beat alignment test
ICC: Intra-class correlation coefficient
GSI: Goldsmith musical sophistication index
MDC: Minimal detectable change
NT: Neurotypical
SEM: Standard error of measurement
